# A case of rectal villous tumor with shortness of breath in a 70‐year‐old woman—Uncommon symptom of McKittrick‐Wheelock syndrome

**DOI:** 10.1002/ccr3.3311

**Published:** 2020-09-08

**Authors:** Konosuke Nakaji, Yuuki Mizuno

**Affiliations:** ^1^ Endoscopy Center Aishinkai Nakae Hospital Wakayama‐shi Japan

**Keywords:** McKittrick‐Wheelock syndrome, rectal villous tumor, shortness of breath, uncommon symptom

## Abstract

Diagnosis of McKittrick‐Wheelock syndrome (MWS) is sometimes difficult because the symptoms are sometimes nonspecific. Physician should be considered MWS in case of severe hyponatremia and hypokalemia are accompanied by prolonged mucous diarrhea.

## Graphical abstract

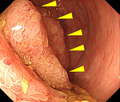

A 70‐year‐old woman with a history of knee osteoarthritis with mucous diarrhea for a year was admitted to our hospital for shortness of breath. Her physical examination and vital signs were normal. Chest computed tomography and cardiac ultrasound were normal. Serum creatinine and blood urea nitrogen were 2.31 and 66.4 mg/dL, respectively. Serum sodium, chloride, and potassium were 135.1, 83.7, and 2.7 mEq/L, respectively. Her endocrine functions including free hyroxine and thyroid‐stimulating hormone were normal. Blood gas analysis revealed pH7.5, PO_2_ 69.2Torr, PCO_2_ 43.5Torr, BE9.4 mEq/l, HCO_3_ 33.7 mEq/L. Abdominal computed tomography showed a large protruded tumor in the rectum. Colonoscopy revealed a mucus‐rich villous tumor in the lower rectum (Figure 1A). Based on these findings, she was diagnosed with McKittrick‐Wheelock syndrome (MWS). With intravenous fluid treatment, laparoscopic low anterior resection was performed. Histological examination of the resected specimens revealed tubular adenocarcinoma in papillary adenocarcinoma which invading the muscularis propria (Figure 1B). Subsequently, her symptom, renal failure, and electrolyte abnormalities improved. Common symptoms of MWS are general fatigue, weakness, and loss of consciousness caused by hyponatremia^1^. Shortness of breath is a rare symptom of MWS, and it was thought to be related to mainly respiratory muscle disorders due to hypokalemia.

**Figure 1:**
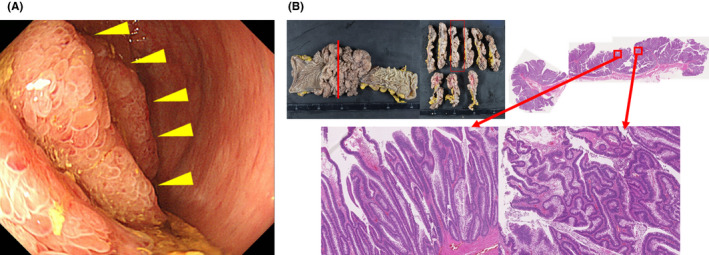
A, Colonoscopy showed a mucus‐rich villous tumor in the lower rectum (yellow arrowheads). B, Histopathological findings of resected specimens showing papillary adenocarcinoma (left red arrow) with tubular adenocarcinoma components (right red arrow) which invading the muscularis propria

## CONFLICT OF INTEREST

None declared.

## AUTHOR CONTRIBUTIONS

MY and NK: took part in the care of the patient. NK: wrote the manuscript. NK: reviewed and supervised the manuscript. All authors approved the final manuscript.

## ETHICAL APPROVAL

Informed consent was obtained from the patient for publication.

## References

[1] Orchard MR , Hooper J , Wright JA , McCarthy K . A systematic review of McKittrick‐Wheelock syndrome. Ann R Coll Surg Engl. 2018;100(8):1‐7. 30322287 10.1308/rcsann.2018.0184 PMC6204505

